# Diversity and Distribution of Archaea Community along a Stratigraphic Permafrost Profile from Qinghai-Tibetan Plateau, China

**DOI:** 10.1155/2014/240817

**Published:** 2014-11-25

**Authors:** Shiping Wei, Hongpeng Cui, Hao He, Fei Hu, Xin Su, Youhai Zhu

**Affiliations:** ^1^School of Marine Sciences, China University of Geosciences, Beijing 100083, China; ^2^Oil and Gas Survey, China Geological Survey, Beijing 100029, China

## Abstract

Accompanying the thawing permafrost expected to result from the climate change, microbial decomposition of the massive amounts of frozen organic carbon stored in permafrost is a potential emission source of greenhouse gases, possibly leading to positive feedbacks to the greenhouse effect. In this study, the community composition of archaea in stratigraphic soils from an alpine permafrost of Qinghai-Tibetan Plateau was investigated. Phylogenic analysis of 16S rRNA sequences revealed that the community was predominantly constituted by *Crenarchaeota* and *Euryarchaeota*. The active layer contained a proportion of *Crenarchaeota* at 51.2%, with the proportion of *Euryarchaeota* at 48.8%, whereas the permafrost contained 41.2% *Crenarchaeota* and 58.8% *Euryarchaeota*, based on 16S rRNA gene sequence analysis. OTU1 and OTU11, affiliated to Group 1.3b/MCG-A within *Crenarchaeota* and the unclassified group within *Euryarchaeota*, respectively, were widely distributed in all sediment layers. However, OTU5 affiliated to Group 1.3b/MCG-A was primarily distributed in the active layers. Sequence analysis of the DGGE bands from the 16S rRNAs of methanogenic archaea showed that the majority of methanogens belonged to *Methanosarcinales* and *Methanomicrobiales* affiliated to *Euryarchaeota* and the uncultured ZC-I cluster affiliated to *Methanosarcinales* distributed in all the depths along the permafrost profile, which indicated a dominant group of methanogens occurring in the cold ecosystems.

## 1. Introduction

Permafrost occupies approximately 25% of Earth's terrestrial surface, occurring most frequently in high latitudes regions, especially those of the Northern Hemisphere [1, 2]. However, alpine permafrost usually exists at low-temperature, high-elevation sites in temperate latitudes. It is well known that permafrost stores massive amounts of carbon; a recent estimate indicates that 1672 Pg of organic carbon, an amount roughly equivalent to the total carbon contained within land plants and the atmosphere [3–5], may exist in the northern permafrost region, which accounts for approximately 50% of the estimated global belowground organic carbon pool [6]. With global warming, the permafrost is starting to thaw, with estimates of as much as 90% of the permafrost being lost by 2100 [7], which raises the question about the fate of carbon in thawing permafrost. It is inferred that release of carbon from permafrost to the atmosphere occurs primarily through accelerated microbial decomposition of organic matter [3]. Previous studies showed that a large variety of microorganisms inhabit permafrost environments [8, 9]. During the thawing, the organic matter becomes more accessible to microbial degradation and results in greenhouse gas emissions [4], which is thought to be one of the most significant feedbacks from terrestrial ecosystems to the atmosphere, thus potentially exacerbating the greenhouse effect and further risking significant climate change [3].

Earlier studies showed that permafrost harbors a diverse microbial community including bacteria and archaea [10–12]. Most studies addressing permafrost microbial community were limited to the sites in Siberian permafrost [13, 14]. A research of Siberian tundra revealed that the total number of bacterial cells from the top to the bottom of the active layer (the top layer of soil that thaws in the summer and refreezes in the winter) range from 2.3 × 10^9^ − 1.2 × 10^8^ cells per gram dry soil [15]. Archaea may constitute between 1% and 12% of the total cells in Siberian active layer soils [15, 16]. The community of archaea was composed of* Euryarchaeota* (61%) and* Crenarchaeota* (39%) in the perennially frozen sediments [11]. Methanogens, affiliated to the phylum of* Euryarchaeota*, are a group of archaea that produce methane under anaerobic conditions, which are ubiquitous in the biosphere and are particularly found in a variety of ecosystems such as rice paddies, lakes, hydrothermal vents, and permafrost soil and sediments [17]. Some of them were proven to be viable inhabitants in the high latitude permafrost [18, 19]. However, the uncultured methanogen cluster, Zoige cluster I (ZC-I), affiliated to* Methanosarcinales* within* Euryarchaeota*, was found to be the dominant group in the alpine permafrost wetland of Tibet plateau [20]. Phylogenetic analyses of the methanogenic* Archaea* community revealed a great diversity of methanogens in the permafrost including families of* Methanobacteriaceae*,* Methanomicrobiaceae*,* Methanosarcinaceae*,* Methanosaetaceae* [16, 17, 19, 20], methanogen group Rice cluster I (RC-I) and uncultured Rice cluster II (RC-II) within the phylogenetic radiation of* Methanosarcinales* and* Methanomicrobiales* [19, 21–23] and ZC-I affiliated to* Methanosarcinales* [20], permafrost cluster I affiliated to* Methanosarcinaceae* [19], and permafrost cluster II and III affiliated to* Methanosarcinales* [19]. The nonmethanogenic archaea Group 1.1b/MCG affiliated to the uncultured* Crenarchaeota* was mostly found in the High Arctic wetland permafrost [24]. Even though we have already accumulated a modest amount of knowledge about the permafrost's methanogenic archaea, they continue to attract the attention of researchers due to their production of methane and the implications for the global warming.

The Qinghai-Tibetan Plateau is the largest alpine permafrost area on Earth. Earlier studies estimated the annual methane emissions from cold wetlands in the Qinghai-Tibetan Plateau at about 0.7–0.9 Tg [25]. With the permafrost thawing due to the greenhouse effect, the organic carbon stored in permafrost would become more accessible for microbes, whose activities will dictate whether permafrost environments will be a net source or sink of greenhouse gas [1]. However, current knowledge of the microbial community in the high-altitude permafrost is poorly understood. In this study, we described the diversity and vertical distribution of archaeal community in the permafrost profile, attempting to elucidate the composition of archaeacolonizing both the active layer and permafrost and to further characterize the methanogen community in Qinghai-Tibetan Plateau.

## 2. Material and Methods

### 2.1. Site Description and Sampling

The sampling site is located in Qinghai-Tibetan Plateau (QTP, N 38°05′38.10′′ and E 99°10′05.13′′) with an elevation of 4300 m above sea level, where summers are mild and winters are quite cold. The mean annual temperature is approximately −5.8°C, with January temperatures ranging from −18 to −7°C and July temperatures ranging from 15 to 21°C. Significant rainfall occurs mainly in summer, while precipitation is very low in winter and spring. The study area belongs to the natural ecosystem of alpine swamp meadows with a large quantity of soil organic carbon storage. The active layer of the permafrost thaws every summer, with the thickness varying from 20 to 60 cm, while the permafrost layer occurs throughout the area to a depth of 600–700 m.

Samples were collected in June, 2013, and were obtained by digging to a depth of 65 cm. Samples were then collected aseptically from the uppermost 3–5 cm of the active layer to 63–65 cm of the permafrost layer at 10 cm intervals. The soil was placed into sterilized plastic bags for chemical analyses and 50 mL centrifuge tubes for microbiological analyses. All the samples were kept frozen in insulated containers and transported immediately to laboratory for further analyses.

### 2.2. Characteristics of the Permafrost Soil

The soil temperature was measured using the in situ method by inserting the pH probe into each layer of the soil. The pH of soil was determined separately on each of the soil samples for each site using a fresh soil to water ratio of 1 : 5 (pH meter, Sartorius PB-10). The water content was determined as the weight loss of fresh soil dried at 105°C for 24 h. Total soil carbon content for each soil sample was determined by combustion for 16 h at 375°C. For analysis of methane concentrations in the soil, 5 g of each sample was taken with a cut-off syringe and injected into a 25 mL bottle containing 10 mL of saturated NaCl solution. The bottles were sealed by a solid butyl rubber septum and shaken for 1 h before they were used. The headspace methane concentration was determined by gas chromatography using a Packard Model 438A fitted with a flame ionization detector.

### 2.3. DNA Isolation

The DNA of the soil samples was extracted using the Soil DNA Isolation Kit (MP) following the manufacturer's instruction. The extracted DNA was visualized on a 1% agarose gel using ethidium bromide staining and quantified by Nanodrop (Thermo Scientiific, NanoDrop ND-2000).

### 2.4. Denaturing Gradient Gel Electrophoresis (DGGE)

A nested-PCR approach was used to amplify V3 region of archaeal 16S rRNA. The full-length archaeal 16S rDNA gene was amplified from genomic DNA using the primer set 8f (5′-CGGTTGATCCTGCCGGA-3′) and 1492r (5′-GGCTACCTTGTTACGACTT-3′) [26]. The first round PCR amplification was performed in 50 *μ*L of reaction mixture containing 0.25 *μ*M of each primer, 0.2 mM dNTP, 1.5 mM MgCl_2_, 5 *μ*L of Taq buffer, 5 U Taq DNA polymerase (Invitrogen, USA), and 10 ng template DNA. The PCR conditions were as follows: 95°C for 5 min, 35 cycles of 50 s at 95°C, 50 s at 45°C, and 1.5 min at 72°C, followed by a final extension for 10 min at 72°C. The PCR products were gel-purified and used as a template to amplify the V3 region by using the primer set 340f-GC (5′-CCCTACGGGGYGCASCAG) and 519r (TTACCGCGGCKGCTG) [26]. The second round PCR conditions were used as follows: 95°C for 5 min, 35 cycles of 50 s at 95°C, 50 s at 45°C, and 1.5 min at 72°C, followed by a final extension for 10 min at 72°C.

DGGE was performed as described by Lazar et al. [26] with modifications. PCR products were applied onto 8% (wt/vol) polyacrylamide gels in 1 × TAE buffer with denaturant gradient from 20 to 40% (100% denaturant consists of 7 M urea and 40% formamide). Electrophoresis was performed at 60°C and 200 V for 6 h. After electrophoresis, the gels were stained with ethidium bromide for 30 min and photographed with a UV imager (AlphaImager Mini System). DGGE bands were gel purified and reamplified with primer set 340f and 519r. The purified PCR products were cloned into vector and transformed into* Escherichia coli* TOP 10 for sequencing.

### 2.5. Clone Library Construction

Archaeal 16S rRNA genes were amplified from soil community DNA using the primers 21f (5′ TTCCGGTTGATCCYGCCGGA) and 958r (5′ YCCGGCGTTGAMTCCAATT); PCR reactions volume was the same with amplification of full length 16S rDNA of archaea, and PCR reactions were carried out according to DeLong [27] with modifications as follows: 1 cycle of 95°C for 1 min, 30 cycles of 94°C for 1 min, 50°C for 1 min, 72°C for 2 min, and a final extension at 72°C for 10 min. The PCR products were purified as described above and cloned following the manufacturer's instructions using the pMD18-T vector system (TaKaRa) together with competent* E. coli* JM109 cells. Randomly selected clones were sequenced.

### 2.6. Sequencing and Phylogenetic Analyses

The sequences were determined on an ABI 3730 automated DNA sequencer (Applied Biosystems) using the universal primer M13-47 or archaeal 21f primer. Sequences of 16S rDNA from DGGE bands and clone libraries were analyzed by using NCBI BLASTN search program to identify their putative closest phylogenetic relatives. The sequences were aligned with their relatives using Clustal W, and phylogenetic trees were constructed by the neighbor-joining method using the maximum-parsimony algorithm in MEG 4 software with 1000 bootstrap replicates. Phylotypes or operational taxonomic units (OTUs) were defined as sequences showing ≥97% homology to each other. Nucleotide sequences have been deposited in the GenBank database and the accession numbers are as follows: DGGE bands, KM251579-KM251601; archaeal clones, KM251602-KM251632.

### 2.7. Statistical Analysis

Clone library coverage was calculated according to Good [28]. A computer program DOTUR was used to assign sequences to OTUs, and then the rarefaction curves were constructed by using the partial 16S rRNA gene sequences [29]. Another computer program MOTHUR was used to calculate the Shannon and Simpson diversity indices, the abundance-based coverage estimator (ACE), and the bias-corrected Chao1 [30].

## 3. Results

### 3.1. Physical and Chemical Characterization of the Permafrost Soil

The samples were collected at the end of June, 2013. The temperature along the soil profile showed a distinct decreasing gradient from the top at 18.34°C to the bottom at −0.45°C. The pH was slightly acidic, ranging from 6.43 to 6.79. Granulometric analysis revealed that the soils in the stratigraphic profile were composed of sand (7.99%–18.73%), silt (66.84%–75.56%), and clay (11.26%–20.49%) with the average grain size ranging from 5.68 to 6.46 *μ*m. Sorting coefficients of the sample varied from 1.73 to 1.99, which indicated that the sediments in the studied sites are well-sorted (Table 1). The water content was highest in the uppermost layer of the soil because of the frequent rainfall in this area during summer months and waterlogging was often observed in the swamp. The organic carbon (TOC) in dried sediment decreased with depth from 9.01% to 1.76%, of which TOC in the topmost layer soil was significantly higher than that of other samples, suggesting an abundant organic matter accumulation in the near surface soil. The methane content was low in the topmost few centimeters but rapidly increased to the maximum value at 188.3 nmol/g in the other deeper samples (Table 1).

### 3.2. Diversity of Archaeal 16S rRNA Gene Sequences in the Clone Libraries

The diversity and composition of archaea community was investigated by constructing clone libraries of archaeal 16S rRNA gene fragments. Seven clone libraries were constructed from the uppermost active soil layer to the lowermost permafrost layer at 10 cm intervals. In each library, the coverage ranged from 95.2% to 97.7% (Table 2). Thirty to fifty-five sequences in each library were retrieved, and a total of 282 sequences were combined for analysis of their diversity and composition in the community. Fifteen operational taxonomic units (OTUs) were identified at the species level (≥97% sequence similarity) through all the depths. Twelve and ten of them were detected in the active layer and the permafrost layer, respectively, and 7 OTUs were presented in both layers. Relatively more OTUs were observed in the depths of 3–5 cm (7 OTUs), 23–25 cm (7 OTUs), and 63–65 cm (OTUs). The Shannon and Simpson diversity indices varied from 0.79 to 1.74 and from 0.18 to 0.58, respectively, and the species richness estimators ACE and Chao 1 varied from 0 to 17 and from 4 to 10, respectively. The Shannon and Simpson diversity indices showed no significant difference, suggesting that diversity of the archaeal 16S rRNA gene libraries was similar among the layers (Table 2).

### 3.3. Composition of Archaeal Communities

**Figure 1:**
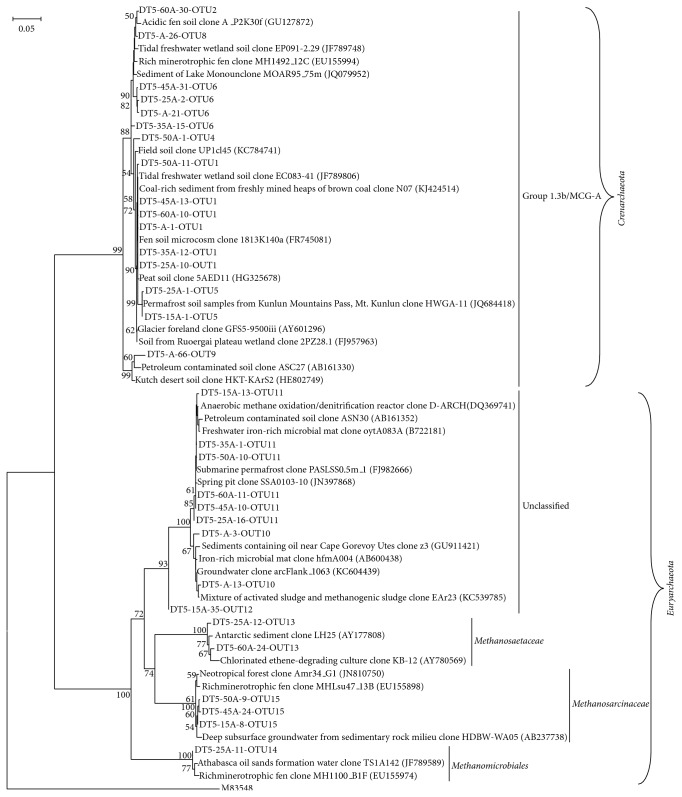
Phylogenetic tree of archaeal 16S rRNA gene sequences from the stratigraphic soil profile. The tree was constructed using the neighbor-joining method from a similarity matrix based on the distance determined by Kimura's two-parameter model. Bootstrap values (100 replications) generated using the maximum parsimony method. The phylotypes were named according to the soil depth origin. Numbers in brackets were GenBank accession numbers. The scale bar represents 5% estimated distance.

Phylogenetic composition of archaeal 16S rRNA gene sequences revealed that they were mainly affiliated to two phyla,* Crenarchaeota* and* Euryarchaeota*, which comprise 47.2%% and 52.8% of the total sequences, respectively. The retrieved crenarchaeal sequences were classified into only one lineage, Group 1.3b/MCG-A [31, 32], which accounted for 47.2% of archaeal clone sequences. The retrieved euryarchaeal sequences could be classified into four lineages,* Methanomicrobiales*,* Methanosarcinaceae*,* Methanosaetaceae*, and unclassified* Euryarchaeota*, which accounted for 0.3%, 1.1%, 1.4%, and 50.0% of archaeal clone sequences, respectively (Figure 1).

**Figure 2:**
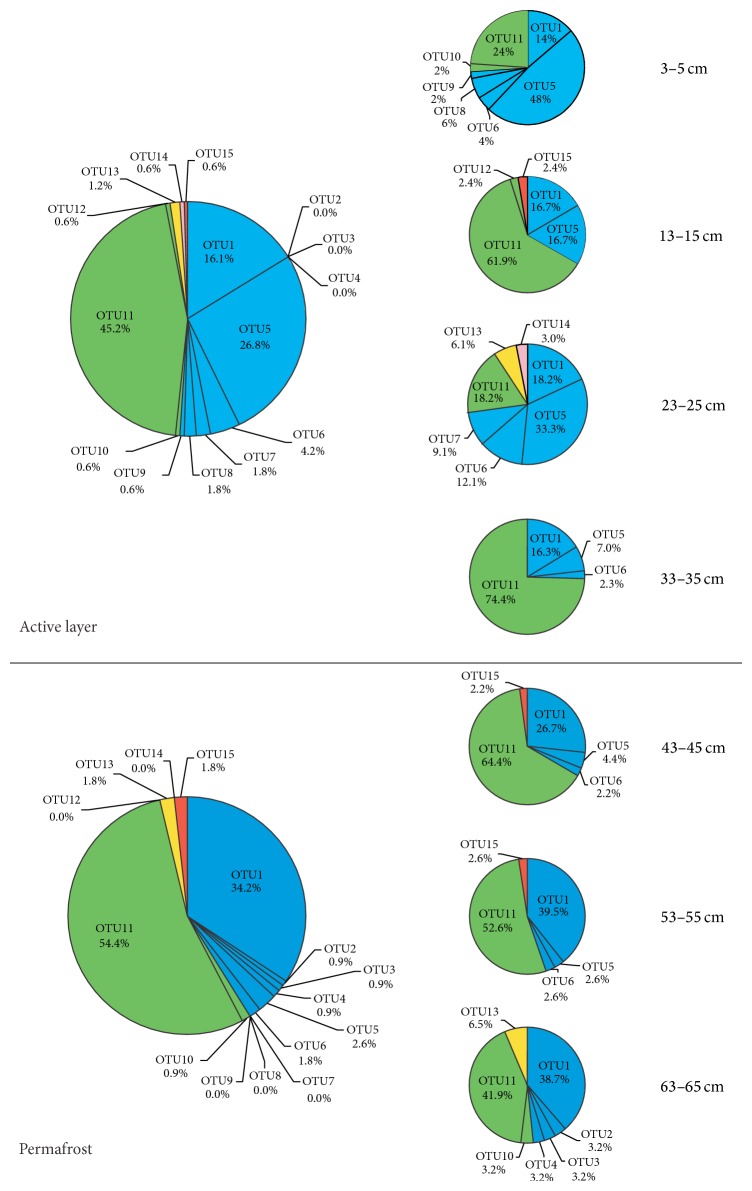
Frequency of individual types (OTU) and relative depth-dependent distribution of groups of the archaea from the stratigraphic permafrost profile based on the gene clone libraries. Green segments indicate OTUs belonging to unclassified* Euryarchaeota*, blue segments indicate OTUs belonging to Group 1.3b/MCG-A, yellow segments indicate OTUs belonging to* Methanosaetaceae*, red segments indicate OTUs belonging to* Methanosarcinaceae*, and pink segments indicate OTUs belonging to* Methanomicrobiales*.

The relative abundance and distribution of different archaeal phylogenetic lineages with the depth were shown in Figure 2. Both active layer and permafrost consisted predominantly of* Crenarchaeota* and* Euryarchaeota*. The active layer contained a proportion of* Crenarchaeota* at 51.2% compared to 48.8%* Euryarchaeota*, whereas the permafrost had a higher proportion of* Euryarchaeota *(58.8%) than* Crenarchaeota* (41.2%). Vertical distribution of OTUs along the sediment profile showed that OTU1 and OTU11, belonging to Group 1.3b/MCG-A and the unclassified group within* Euryarchaeota*, respectively, were widely distributed in all sediment layers. The abundance of OTU1 was gradually increased with depths, whereas OTU11 gradually decreased with depths except for the topmost three soil layers. OTU5, another lineage belonging to Group 1.3b/MCG-A within* Crenarchaeota*, was predominantly distributed only in the top parts of the active layers. It was very interesting that the occurrence of OTU5 was reduced sharply with depths, especially when the temperature dropped below 0.4°C; we inferred that OTU5 was probably controlled by the low temperatures. Three OTUs (OTU2, OTU3, and OTU4) were only detected in the permafrost layer and were not found in the active layer. Conversely, distributions of OTU7, OTU8, OTU9, OTU12, and OTU14 were only detected in the active layers, which suggested that the communities of archaea varied over the different depths.

### 3.4. Denaturing Gradient Gel Electrophoresis Analysis of 16S rRNA of Methanogenic Archaea

**Figure 3:**
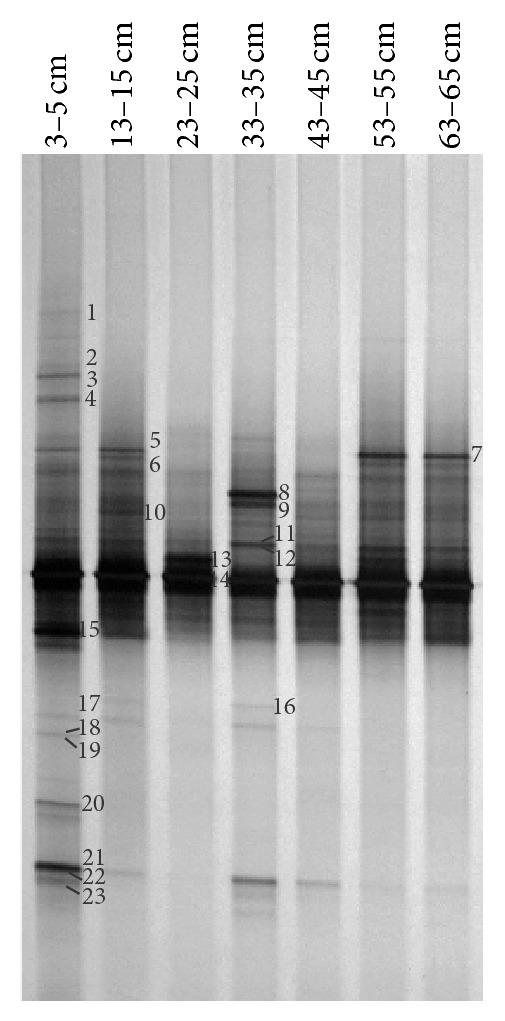
DGGE of PCR-amplified archaeal 16S rRNA gene fragments from total community DNA from different depths (indicated above the lanes) in the soil profile. Marked DGGE bands were excised and sequenced. The numbers of DGGE bands correspond to the ones on the phylogenetic tree in Figure 4.

**Figure 4:**
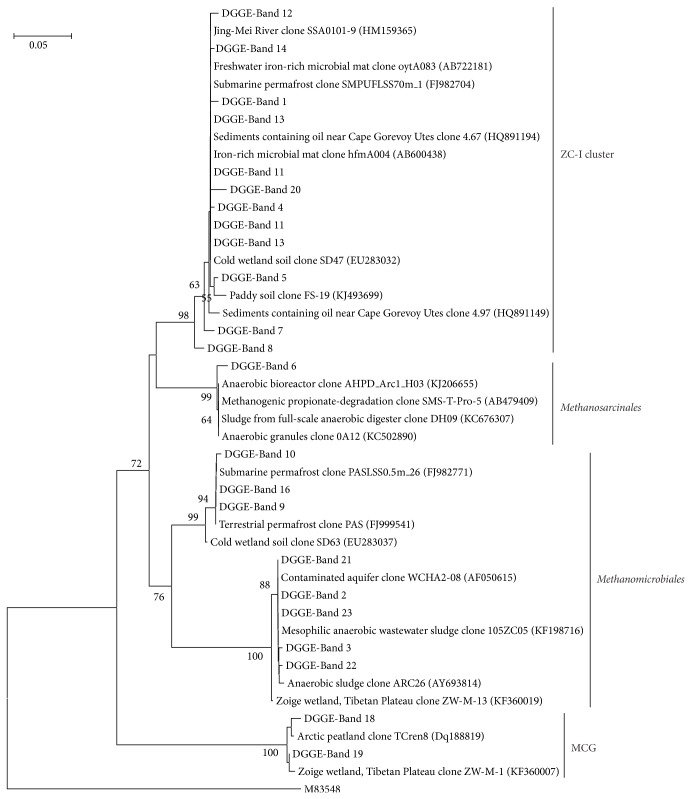
Phylogenetic relationships of archaeal 16S rRNA gene sequences obtained from DGGE bands in the soil profile. The scale bar represents 5% estimated distance.

Denaturing gradient gel electrophoresis (DGGE) fingerprinting was used to characterize the community composition of methanogenic archaea. DGGE band patterns indicated that most DNA bands were observed in the topmost layer of the profile (3–5 cm), and similar band patterns were observed at all the depths below, which indicated the occurrence of similar methanogenic archaea communities in the lower layers of the profile. Major bands with strong intensity were commonly observed in all depths, which suggested that dominant DGGE bands represented the abundant methanogenic archaea in the community. However, several DGGE bands detected in the different layers of soil were not present in other layers, indicating depth-specific methanogenic populations. A total of 23 bands were excised and sequenced. Phylogenetic analysis showed that twelve sequences fell within the euryarchaeotal lineage, comprising three groups of ZC-I cluster (12 sequences),* Methanosarcinales* (1 sequence), and* Methanomicrobiales* (8 sequences). Only two sequences were retrieved in the Miscellaneous Crenarchaeal Group (MCG) under crenarchaeotal lineage. Sequences affiliated to ZC-I cluster were distributed in all depths, whereas sequences associated with* Methanomicrobiales* were mainly distributed in the top and middle of the permafrost sediments, but the sequences affiliated to MCG were only observed in the topmost active layer of permafrost. It was very interesting that only two bands affiliated to ZC-I cluster were observed at the depth of 23–25 cm, which showed the maximum methane concentration (Figures 3 and 4 and Table 1).

## 4. Discussion

Permafrost is well-known for storing massive amounts of organic carbon and is thought to be the most important natural methane emission source [3, 33]. Previous studies estimated that a total of 33.52 Pg of organic carbon and 0.7–0.9 Tg of methane emission were emitted from the Qinghai-Tibetan Plateau grassland soils [25, 34]. Recent studies indicated a continuous increase in air temperatures on the QTP over the last 40 years, making them a potential source of considerable greenhouse gas emissions [35]. Our data showed that the organic carbon content ranged from 1.61% to 3.02% at depths of 13–65 cm, which is comparable with 1.6% in a high Arctic permafrost soil from Spitsbergen reported by Hansen et al. [8] and 1.2–3.0% in a permafrost active layer soil from Lena Delta reported by Liebner and Wagner [13]. However, the organic carbon content reached 9.01% in the topmost soil (3–5 cm) layers of our QTP sites. The higher organic carbon was inferred to be related to the decayed grass roots in the wetland. The methane content was much lower in both the top and bottom sediments along the permafrost profile except the subsurface at depth of 23–25 cm, where the methane content could reach a maximum value at 188.3 nmol/g. Patterns of methane content did not exhibit any trends with depth nor correlate noticeably with the physical and chemical properties of the sediments, such as organic carbon concentration, sedimentary properties, or water content. This observation was consistent with that Rivkina et al. [36] reported in the north-eastern Arctic tundra. The higher methane content at depth of 23–25 cm was inferred to be related with the lower redox potential and suitable substrates in this layer, which needs to be further proved.

A total of 15 OTUs of archaea were observed from the Qinghai-Tibetan Plateau permafrost soil, which presented a lower archaeal diversity when compared with other environments. Steven et al. [9] revealed 43 OTUs of archaea in a permafrost/ground ice core from the Canadian High Arctic. The lower diversity of archaea in QTP permafrost soil may be related to the habitat environments, which did not vary greatly as evidenced by the similar physiochemical variables shown in Table 1. Frank-Fahle et al. [37] reported that microbial diversity was highest in the surface layers and decreased towards the permafrost layers. Our data showed that the diversity of the archaea was similar among the layers and lower overall OTU numbers in each layer were observed. Existence of overlapping OTUs of archaeal communities from different depths suggested that the dominant archaeal community was similar over the sampling range.

The communtiy composition revealed by 16S rRNA gene clone libraries showed that* Crenarchaeota* and* Euryarchaeota* were the dominant phyla in both active layer and permafrost. A similar abundance of* Crenarchaeota* (51.2%) and* Euryarchaeota* (48.8%) was observed in the active layer. In contrast, a little higher abundance of* Euryarchaeota* (58.8%) and a little lower abundance of* Crenarchaeota* (41.2%) were observed in the permafrost layer (Figure 2). This result is quite similar with the report by Steven et al. [9] from the Canadian High Arctic, in which the 16S rRNA gene sequences belonging to the* Crenarchaeota* dominated the active layer and permafrost table horizons, while* Euryarchaeota* were predominant in the permafrost. However, Wilhelm et al. [24] reported that both active layer and permafrost consisted predominantly of* Crenarchaeota* at 71% and 95%, respectively; the active layer had a greater proportion of* Euryarchaeota* (22%) compared with permafrost (4%). The abundance of* Crenarchaeota* was inferred to be an increasing trend with a decreasing temperature, which suggested that members of* Crenarchaeota* may be particularly adapted to cold conditions [16]. Nevertheless, our data indicated that even though both OTU1 and OTU5 belong to Group 1.3b/MCG-A within* Crenarchaeota*; 16.2% of OTU1 and 26.8% of OTU5 were distributed in the active layer; in comparison, 34.2% of OTU1 and 3.5% of OTU5 were distributed in the permafrost (Figure 2), suggesting that OTU1 may be more adapted to the cold environment whereas OTU5 appears more cold sensitive.

Based on 16S rRNA gene sequence analysis, both* Crenarchaeota* and* Euryarchaeota* showed a limited diversity with only one group (Group 1.3b/MCG-A) underlying* Crenarchaeota* and four groups (*Methanomicrobiales*,* Methanosarcinaceae*,* Methanosaetaceae*, and an unclassified group) underlying* Euryarchaeota*. A total of 97.2% sequences belonged to both Group 1.3b/MCG-A (47.2%) and unclassified* Euryarchaeota* (50.0%), which were predominantly comprised of OTU11 (48.9%) and OTU1 (23.4%), respectively (Figure 2). Ochsenreiter et al. [31] reported that Group 1.3b, an noncultured group of* Crenarchaeota*, was widely distributed in different environments such as freshwater, wastewater, and soil. Sequences of the 16S rRNA gene affiliated to unclassified group within noncultured* Euryarchaeota* were frequently present in freshwater environments. Our data showed that Group 1.3b/MCG-A and unclassified* Euryarchaeota* were predominant archaea distributed over all the depths. It was very interesting that OTU5 affiliated to Group 1.3b/MCG-A was overwhelmingly distributed in the active layers, and sequences belonging to OTU1 tended to increase gradually with depth, while sequences belonging to OTU11, affiliated to unclassified* Euryarchaeota*, were primarily distributed in the layers with much lower tempertures (Figure 2). To our knowledge, those findings have never been reported previously.

Methanogenic archaea, also known as methanogens, are an important group of* Euryarchaeota* that produce methane under anaerobic conditions and have proven to be viable inhabitants of permafrost [36, 38]. Our DGGE sequences analyses revealed that members of* Methanosarcinales* and* Methanomicrobiales* constituted the majority of methanogens in the soils, and the uncultured methanogen of ZC-I cluster affiliated to* Methanosarcinales* showed the strongest intensity of bands in DGGE and was distributed in all the depths along the permafrost profile, which indicated a dominant group of methanogens occurring in cold ecosystems. Similar observation was reported in the Zoige wetland of the Tibetan Plateau [20].* Methanosarcinales* are widespread in diverse anaerobic habitats including freshwater and marine mud and sediments, rumens, and sewage sludge digestors [39]. They are capable of using acetate as a substrate for methanogenesis, distinguishing them from* Methanomicrobiales*, which can use H_2_ and CO_2_ as a substrate for methanogenesis [40]. Our data showed that members of* Methanomicrobiales* generally showed less intense bands in DGGE and were distributed in limited layers, which indicated that the acetotrophic methanogens were relatively more abundant than the hydrogenotrophic methnogens in the permafrost. Chin and Conrad [41] reported when a paddy soil shifts to a low temperature, it resulted in a transient accumulation of acetate. Another report from Wagner and Pfeiffer [42] showed that acetate usually serves as the substrate for methanogenesis at a lower temperature. The distribution pattern of methanogens in QTP permafrost is consistent with a report from a Siberian Arctic permafrost in which* Methanosarcinales* were the dominant methanogens in the low temperature environment [19]. Using the DGGE primers for amplification of methanogenic archaea, we recovered two sequences affiliated to MCG within* Crenarchaeota* from the topmost layer of the active permafrost, where the organic carbon content is very high (9.01%). The MCG archaea, a group of heterotrophic anaerobes, have a much wider habitat range that includes terrestrial and marine, hot and cold, and surface and subsurface environments [43, 44]. Currently, the carbon and energy sources for the MCG are unknown. Previously researches indicated that they were usually dominant in nutrient-rich environments and may utilize complex organic substrates [44–47].

## 5. Conclusion

In this study, we have investigated the archaeal community composition in stratigraphic soils from an alpine permafrost of Qinghai-Tibetan Plateau. Diversity of archaea was similar among all the depths, and the community was predominantly constituted by* Crenarchaeota* and* Euryarchaeota*. Each group of those two phyla has a unique distribution pattern between the active layers and the permafrost layers. The majority of methanogens belonged to* Methanosarcinales* and* Methanomicrobiales* under the phyla of* Euryarchaeota*. The present study will help improve our understanding of the community structure of archaea in the permafrost environment.

## Figures and Tables

**Table 1:** Physiochemical properties of soil samples at different depths.

Soil depth (cm)	Temperature (°C)	pH	Sand (%)	Silt (%)	Clay (%)	Average grain size (*µ*m)	Standard deviation (*σ*)	Water content (%)	TOC (%)	CH_4_ (nmol/g)
3–5	18.34	6.79	16.33	72.40	11.26	5.68	1.80	46.52	9.01	4.1
13–15	3.68	6.70	7.99	75.56	16.45	6.25	1.73	25.56	3.02	19.9
23–25	1.85	6.74	13.19	69.81	17.00	6.18	1.91	21.35	2.43	188.3
33–35	0.44	6.60	8.06	71.45	20.49	6.46	1.82	17.18	2.43	79.3
43–45	−0.12	6.44	10.85	69.79	19.36	6.30	1.90	19.23	1.76	6.7
53–55	−0.24	6.57	18.73	66.84	14.43	5.84	1.99	20.91	1.61	22.5
63–65	−0.45	6.43	15.00	68.96	16.05	6.06	1.94	25.54	1.96	16.1

**Table 2:** Sequencing information and statistical analyses of archaeal 16S rRNA gene clone libraries.

Soil depth	NC	NO	Coverage (%)	ACE	Chao 1	Shannon	Simpson
3–5 cm	50	7	96.0	9.24 (7.3–24.6)	7.5 (7.0–15.3)	1.42 (1.2–1.7)	0.30 (0.21–0.40)
13–15 cm	42	5	95.2	6.94 (5.2–24.2)	6 (5.1–18.5)	1.07 (0.8–1.3)	0.43 (0.29–0.56)
23–25 cm	33	7	97.0	7 .38 (7.0–12.2)	7 (0–7)	1.74 (1.5–2.0)	0.18 (0.11–0.25)
33–35 cm	43	4	97.7	4.78 (4.1–14.0)	4 (0–4)	0.79 (0.5–1.1)	0.58 (0.41–0.74)
43–45 cm	45	5	95.6	8 (5.6–20.8)	5.5 (5.0–13.3)	0.94 (0.7–1.2)	0.48 (0.35–0.60)
53–55 cm	39	5	97.4	0 (0-0)	8 (5.4–29.4)	0.99 (0.7–1.3)	0.42 (0.33–0.51)
63–65 cm	30	7	96.7	17 (8.1–98.3)	10 (7.4–30.0)	1.35 (1.0–1.7)	0.31 (0.21–0.41)

Notes: NC is the number of clones in each library. NO is the number of Operational Taxonomic Unit (OTU) based on 97% nucleotide identity.
